# Multiple microRNAs regulate human *FOXP2* gene expression by targeting sequences in its 3′ untranslated region

**DOI:** 10.1186/s13041-014-0071-0

**Published:** 2014-10-01

**Authors:** Lijuan Fu, Zhimin Shi, Guanzheng Luo, Weihong Tu, XiuJie Wang, Zhide Fang, XiaoChing Li

**Affiliations:** Neuroscience Center of Excellence, LSU Health Sciences Center, New Orleans, LA 70112 USA; Key Laboratory of Genetic Network Biology, Institute of Genetics and Developmental Biology, Chinese Academy of Sciences, Beijing, 100101 China; Biostatistics Program, LSU Health Sciences Center, New Orleans, LA 70112 USA

**Keywords:** miRNAs, *FOXP2*, 3′ UTR, Post-transcriptional regulation, Speech and language, Cerebellum

## Abstract

**Background:**

Mutations in the human *FOXP2* gene cause speech and language impairments. The FOXP2 protein is a transcription factor that regulates the expression of many downstream genes, which may have important roles in nervous system development and function. An adequate amount of functional FOXP2 protein is thought to be critical for the proper development of the neural circuitry underlying speech and language. However, how *FOXP2* gene expression is regulated is not clearly understood. The *FOXP2* mRNA has an approximately 4-kb-long 3′ untranslated region (3′ UTR), twice as long as its protein coding region, indicating that *FOXP2* can be regulated by microRNAs (miRNAs).

**Findings:**

We identified multiple miRNAs that regulate the expression of the human *FOXP2* gene using sequence analysis and *in vitro* cell systems. Focusing on let-7a, miR-9, and miR-129-5p, three brain-enriched miRNAs, we show that these miRNAs regulate human *FOXP2* expression in a dosage-dependent manner and target specific sequences in the *FOXP2* 3′ UTR. We further show that these three miRNAs are expressed in the cerebellum of the human fetal brain, where *FOXP2* is known to be expressed.

**Conclusions:**

Our results reveal novel regulatory functions of the human *FOXP2* 3′ UTR sequence and regulatory interactions between multiple miRNAs and the human *FOXP2* gene. The expression of let-7a, miR-9, and miR-129-5p in the human fetal cerebellum is consistent with their roles in regulating *FOXP2* expression during early cerebellum development. These results suggest that various genetic and environmental factors may contribute to speech and language development and related neural developmental disorders via the miRNA-*FOXP2* regulatory network.

**Electronic supplementary material:**

The online version of this article (doi:10.1186/s13041-014-0071-0) contains supplementary material, which is available to authorized users.

## Background

Dysfunctions of the human *FOXP2* gene have been implicated in speech and language impairments [1-6]. As a transcription factor, the FOXP2 protein controls the expression of hundreds of downstream genes, many of which play important roles in nervous system development and function [7-11]. An adequate amount of functional FOXP2 protein is thought to be critical for the proper development of the distributed neural circuits underlying speech and language [12,13]. However, how the expression of the human *FOXP2* gene is regulated is not clearly understood. miRNAs are small nonprotein-coding RNA molecules that regulate gene expression post-transcriptionally by targeting specific sequences in the 3′-UTRs of mRNAs, leading to mRNA degradation and/or translational suppression [14,15]. The *FOXP2* mRNA has an approximately 4-kb long 3′ UTR, twice as long as its protein coding region [1], raising the possibility that *FOXP2* expression is regulated by miRNAs. Here we report the identification of multiple miRNAs that downregulate the expression of the human *FOXP2* gene by targeting specific sequences in its 3′ UTR.

## Results

### Identification of miRNA binding sites in the human *FOXP2* 3′ UTR

**Figure 1:**
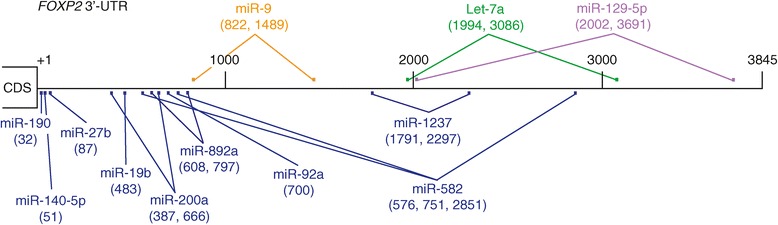
**Predicted miRNA binding sites in the human** ***FOXP2*** **3′ UTR.** The numbers in the parentheses indicate the positions of miRNA binding sites in the human *FOXP2* 3′ UTR. The first nucleotide downstream from the stop codon is denoted as +1.

Using cDNAs made from human fetal brains combined with polymerase chain reaction (PCR), we cloned a 3845 nt sequence fragment that matched 100% to the human *FOXP2* 3′ UTR sequence annotated in the NCBI database. We searched this *FOXP2* 3′ UTR sequence for miRNA binding sites, requiring a perfect match to the 7-nt seed sequence in a miRNA [15,16]. This search predicted numerous miRNAs that potentially target the *FOXP2* 3′ UTR. Some of these miRNAs have more than one binding site and the binding site sequences are conserved in higher vertebrate species. We selected 12 miRNAs: miR-9, miR-19b, miR-27b, miR-92a, miR-140-5p, miR-190, miR-200a, let-7a, miR-129-5p, miR-582-5p, miR-892a, and miR-1237 (Figure 1) and tested whether they downregulate *FOXP2* expression in cell culture systems.

### Multiple miRNAs downregulate human FOXP2 protein and mRNA expression

**Figure 2:**
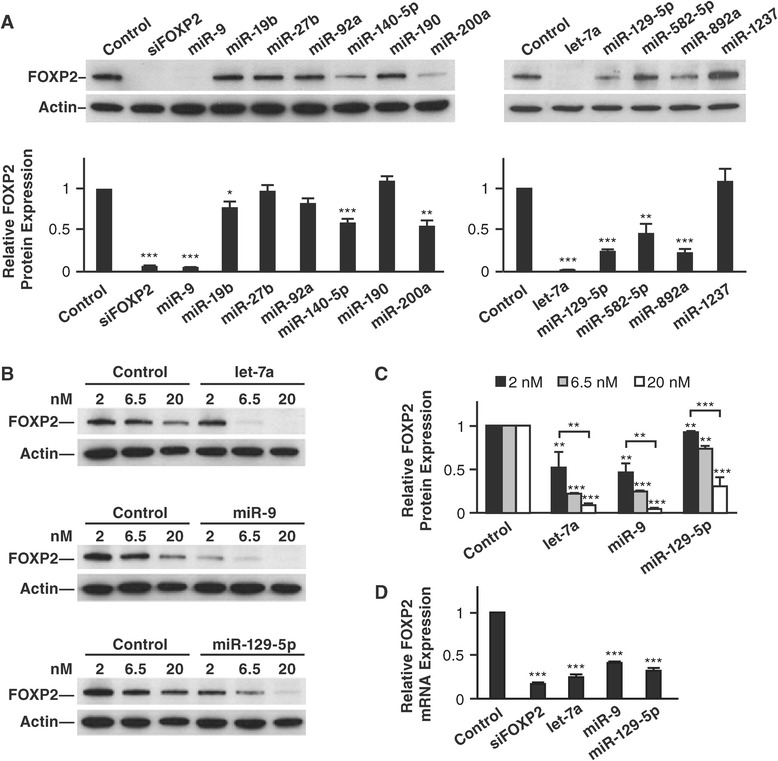
**Multiple miRNAs downregulate human FOXP2 expression. (A)** Representative Western blot images and quantitative results showing that miR-9, miR-19b, miR-140-5p, miR-200a, let-7a, miR-129-5p, miR-582-5p, and miR-892a downregulated human FOXP2 protein expression when transfected into HEK293 cells. **(B and C)** The downregulatory effects of let-7a, miR-9, and miR-129-5p were dosage-dependent. **(D)** Let-7a, miR-9, and miR-129-5p downregulated *FOXP2* mRNA expression in similar transfection experiments as measured by qRT-PCR. For both Western blot and qRT-PCR, Actin was used as an internal control; a miRNA mimic with a scrambled sequence (Ambion) was used as a negative control; and a siRNA against *FOXP2* siFOXP2 was used as a positive control. Transfection was performed at least three times (n = 3) for each miRNA, and quantifications were averaged from the three transfections, two Western blot or two qRT-PCR experiments per transfection. *p < 0.05, **p < 0.01, and ***p < 0.001.

We transfected mimics of each candidate miRNA into human HEK293 cells (where *FOXP2* is expressed endogenously) and measured FOXP2 protein levels 72 hours later by Western blot analysis. We found that miR-9, miR-19b, miR-140-5p, miR-200a, let-7a, miR-129-5p, miR-582-5p, and miR-892a reduced FOXP2 protein levels significantly (Figure 2A). Of these miRNAs, let-7a, miR-9, and miR-129-5p were among the most effective regulators, reducing FOXP2 protein by 70-90%; they are also known to be abundantly expressed in vertebrate brains [17,18]. We focused on these three miRNAs and tested whether their regulatory effects were dose-dependent at three dosages: 2, 6.5, and 20 nM. At 2 nM, comparing to the control, let-7a and miR-9 each decreased FOXP2 protein levels by about 50%, while miR-129-5p decreased FOXP2 protein by less than 10% (*p* < 0.01 for all). At 20 nM, let-7a and miR-9 decreased FOXP2 protein levels by 90%, and miR-129-5p decreased FOXP2 protein by 70% (*p* < 0.001 for all, Figure 2B and C). The dose-dependent downregulation was significant for each miRNA (*p* < 0.0018 for let-7a and miR-9; *p* < 0.0006 for miR-129-5p, Jonckheere-Terpstra test). Using quantitative real time PCR (qRT-PCR), we also found that *FOXP2* mRNA level was downregulated by let-7a, miR-9, and miR-129-5p in similar transfection experiments (Figure 2D).

### let-7a, miR-9, and miR-129-5p target specific sequences in the human *FOXP2* 3′ UTR

**Figure 3:**
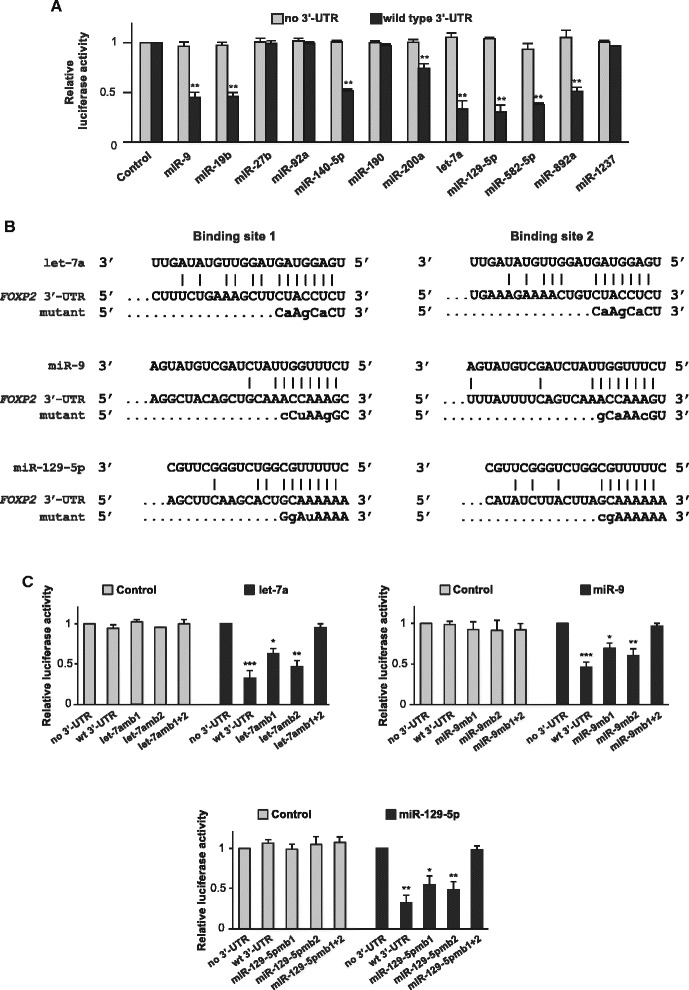
**The downregulatory effects of let-7a, miR-9, and miR-129-5p are mediated via specific sequences in the human** ***FOXP2*** **3′ UTR. (A)** miR-9, miR-19b, miR-140-5p, miR-200a, let-7a, miR-129-5p, miR-582-5p, and miR-892a significantly repressed luciferase activity when co-transfected with a reporter construct containing a wild-type *FOXP2* 3′ UTR, but had no effects when co-transfected with a reporter construct containing no 3′ UTR. **(B)** Mutations in the binding sites for let-7a, miR-9, and miR-129-5p in the human *FOXP2* 3′ UTR. Mutated nucleotides are in lower case letters. **(C)** Mutations in both the two binding sites for let-7a, miR-9, and miR-129-5p in the *FOXP2* 3′ UTR abolished the repressive effects of these miRNAs in luciferase reporter assays. Transfection was performed three times (n = 3) for each miRNA, and luciferase assays were performed in duplicate. To control for transfection efficiency, *Renilla* luciferase activity was normalized to the internal control firefly luciferase activity. Error bars = SEM; *p < 0.05, **p < 0.01, and ***p < 0.001, Tukey’s “Honest Significant Difference” test.

Next, we tested whether the regulatory effects of candidate miRNAs were mediated by the 3′ UTR of the human *FOXP2* gene using luciferase reporter assays. We co-transfected miRNA mimics and a luciferase reporter construct containing a wild type *FOXP2* 3′ UTR or no 3′ UTR into SH-SY5Y cells, where no endogenous FOXP2 was expressed, and performed luciferase reporter assays. Compared to a control miRNA with scrambled sequence, the eight candidate miRNAs, miR-9, miR-19b, miR-140-5p, miR-200a, let-7a, miR-129-5p, miR-582-5p, and miR-892a significantly repressed luciferase activity when co-transfected with a reporter construct containing a wild type *FOXP2* 3′ UTR (*p* < 0.01 for all), but had no effect when co-transfected with a reporter construct containing no *FOXP2* 3′ UTR. In contrast, miR-27b, miR-92a, miR-190, and miR-1237 did not repress luciferase activity whether the reporter constructs contained a *FOXP2* 3′ UTR or not (Figure 3A). These results were consistent with the Western blot results obtained from HEK293 cells as described in Figure 2A, indicating that the regulatory functions of these miRNAs were independent of the cell lines we used.

We focused on let-7a, miR-9, and miR-129-5p and further tested whether their regulatory effects were sequence-specific. Each of these miRNAs has two binding sites in the human *FOXP2* 3′ UTR. For each miRNA, we made three mutant luciferase reporter constructs with sequences in binding site 1, binding site 2, and binding sites 1 + 2 mutated respectively by site-directed mutagenesis (Figure 3B). Using luciferase reporter assays, we showed that each of these miRNAs significantly repressed luciferase activity when only one binding site was mutated (*p* < 0.05 or *p* < 0.01 for all three miRNAs), but their regulatory effects were completely abolished when both binding sites were mutated (*p* > 0.98 for all three miRNAs, Figure 3C). In comparison, the negative control miRNA had no effect on all reporter constructs whether it had no 3′ UTR, wild type 3′ UTR, or mutated 3′ UTRs (Figure 3C). These results suggested that the downregulatory effects of these miRNAs were mediated through specific sequences in the *FOXP2* 3′ UTR, and each of their respective two binding sites was functional.

### Let-7a, miR-9, and miR-129-5p are expressed in the cerebellum of the human fetal brain

**Figure 4:**
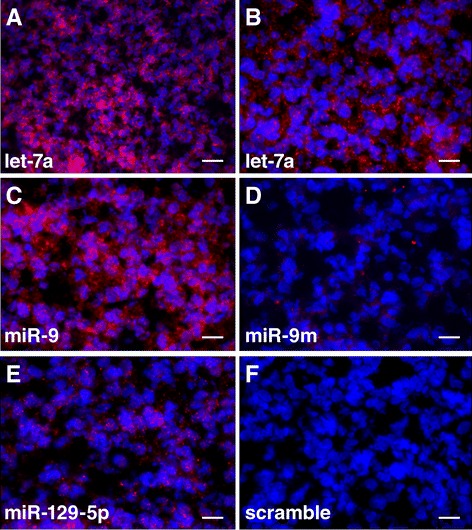
**Expression of let-7a, miR-9, and miR-129-5p in the cerebellum of the human fetal brain.** In all images, miRNA hybridization signals are in red (Cy3) and DAPI stained cell nuclei are in blue. **(A and B)** A cerebellum section hybridized with a LNA modified let-7a detection probe. Note the cytoplasmic localization of the let-7a signal. **(C)** A cerebellum section hybridized with a miR-9 detection probe. **(D)** A miR-9 probe with 4 nucleotides mutated (miR-9m) detected no hybridization signal. **(E)** A cerebellum section hybridized with a miR-129-5p detection probe. **(F)** A negative control probe with scrambled sequence detected no hybridization signal. The scale bars are 40 μm in A and 20 μm in B through F.

*FOXP2* is known to be expressed in several brain regions in the human fetal brain, including the cerebellum, the basal ganglia, the thalamus, and the cortical plate [19]. miRNAs that regulate *FOXP2* expression and play a role in brain development are expected to be expressed in similar brain regions. We examined the expression of let-7a, miR-9, and miR-129-5p in human fetal brain tissue by *in situ* hybridization using Locked Nucleic Acid (LNA) modified miRNA detection probes. Due to limited tissue availability, only the cerebellum (16 weeks old) was examined. We found that all these three miRNAs, let-7a, miR-9, and miR-129-5p, were expressed in the cerebellum of the human fetal brain (Figure 4). These observations were consistent with the roles of these miRNAs in regulating *FOXP2* expression during human cerebellum development.

## Discussion

A heterozygous missense mutation in the human *FOXP2* protein coding region (i.e., R553H) results in a transcription factor with compromised DNA binding and transcriptional regulatory activity [1,20]. Presumably, reduced functional dosage of cellular FOXP2 protein causes speech and language impairments [12]. Our results, together with recent reports that miR-9, miR-132, and miR-140-5p regulate *Foxp2*/*FoxP2* expression (*Foxp2* and *FoxP2* denote respective rodent and avian genes) in animal models [21,22], highlight the importance of the *FOXP2* 3′ UTR sequence and the roles for miRNAs in regulating *FOXP2* expression. These results raise the possibility that sequence variations in the *FOXP2* 3′ UTR, particularly in miRNA binding sites, due to mutations and/or polymorphism may contribute to dysregulation and/or functional variations of the *FOXP2* gene. In addition, various genetic, physiological, and environmental factors may influence the expression of miRNAs, thus indirectly impacting *FOXP2* expression.

Human subjects carrying *FOXP2* mutations exhibit structural and functional abnormalities, presumably due to FOXP2 malfunction, in several brain regions, including the cerebellum [23], where *FOXP2* is known to be expressed [19]. The expression of let-7a, miR-9, and miR-129-5p in the human fetal cerebellum is consistent with their roles in regulating *FOXP2* expression during early cerebellum development in humans. Recently, dysregulation of miR-129-5p is found in the cerebellum of autistic brains, albeit only a limited number of brains were examined [24]. Further investigation of *in vivo* functions of these miRNAs will bring insights into their roles in speech and language development and related neural developmental disorders.

## Methods

### Cloning of the human *FOXP2* 3′ UTR and prediction of miRNA binding sites

Human fetal brain poly(A)+RNA (Clontech) was reverse-transcribed into cDNA using an oligo-dT primer. Using primers designed based on the human *FOXP2* 3′ UTR sequence in the NCBI database [NCBI: NM148898, variant II], we PCR amplified two overlapping fragments representing the *FOXP2* 3′ UTR. The two fragments were ligated at a BamH1 site to obtain a 3845-nt full length human *FOXP2* 3′ UTR. This sequence was used for miRNA binding sites prediction using the software TargetScan. Sequences in the *FOXP2* 3′ UTR matching perfectly to the 7-nt seed sequence of a miRNA were accepted as putative miRNA binding sites [16].

### Transfection, Western blotting, and qRT-PCR

miRNA mimics (Ambion) were transfected into human HEK293 cells (60 nM) using Lipofectamine 2000 (Invitrogen) and cells were harvested 72 hours later for protein and mRNA assays. Proteins were separated by electrophoresis on 10% SDS–PAGE gel. After blotting, membranes were probed with an antibody against the human FOXP2 protein (sc-21069, Santa Cruz Biotechnology). Protein bands were visualized using ECL-Plus and quantified with Image J. For qRT-PCR, total RNA was extracted from transfected cells and reverse-transcribed (iScript cDNA synthesis kit, Bio-Rad). qRT-PCR was performed using the SYBR Green Supermix (Bio-Rad). Primer sequences are listed in the Additional file 1: Figure S1. The specificity of PCR amplification products were validated by electrophoresis and the melting-curve analysis. For each miRNA, transfection was performed at least three times. For each transfection, Western blot and qRT-PCT were performed at least two times, and qRT-PCR was performed in triplicate.

### Luciferase reporter assays

Plasmid constructs carrying wild-type or mutant human *FOXP2* 3′ UTRs in the psiCHECK vector (Promega, 100 ng) were co-transfected with miRNA mimics (100 nM) into SH-SY5Y cells. Cells were harvested 48 h later and luciferase activity was assayed using the Dual-Luciferase Reporter System (Promega). To control for transfection efficiency, *Renilla* luciferase activity was normalized to the internal control firefly luciferase activity. For each miRNA, transfection was performed three times and luciferase activity was assayed in duplicate.

### Mutagenesis of miRNA binding sites

The full length human *FOXP2* 3′ UTR sequence was used as a template in site-directed mutagenesis of miRNA binding sites using the QuikChange II XL Mutagenesis Kit (Agilent). Specific PCR primers were designed using the QuikChangePrimer Design tool (see Additional file 1: Figure S1) and synthesized by Integrated Device Technology (IDT: http://www.idt.com). In some cases, to mutate three nucleotides in a binding site, we performed 2 rounds of mutagenesis. For example, we mutated 2 nucleotides in the first round, and used the mutated plasmid as a template in the second round to mutate the third nucleotide. All mutated binding sites were sequence verified.

### *In situ* hybridization

*In situ* hybridization was performed as described previously [22]. Briefly, fixed brain sections (10 μm thick) were hybridized with LNA modified miRNA detection probes at 38-45°C overnight. After washing and blocking, slides were incubated with an Anti-Digoxigenin antibody for 1 h, followed by signal amplification using the TSA Plus Cy3 System (PerkinElmer). LNA modified miRNA detection probes were purchased from Exiqon: let-7a probe (18000–01); miR-9 probe (88078–05); a customer designed mutant miR-9 probe (miR-9m: 5′-TCATA**G**AGCTA**C**ATAACCA**T**A**C**A-3′, underlined are mutated nucleotides); miR-129-5p probe (38482–15); negative control probe (99004–01). The human fetal brain (16 weeks old) cerebellum sections were obtained from a commercial source (Biochain Institute, CA).

## Additional file

Additional file 1: Figure S1. Sequences of primers used in respective experiments.

## Abbreviations

3′ UTRs: 3′ untranslated regions
qRT-PCR: Quantitative real time polymerase chain reaction
