# Supplementary material for: Multiple microRNAs regulate human FOXP2 gene expression by targeting sequences in its 3′ untranslated region
Source: Mol Brain. 2014 Oct 1;7:71. doi: 10.1186/s13041-014-0071-0 (PMC4189591; doi:10.1186/s13041-014-0071-0)
Supplement: Additional file 1: Figure S1. — Sequences of primers used in respective experiments. [file 13041_2014_71_MOESM1_ESM.pdf]

Additional Figure S1

Primers used for human *FOXP2* 3'-UTR cloning

Fragment 1

forward primer: 5'-CA<u>GCGATCGC</u>GAACTGACTTGTGAAACCTCAGCG-3'

(a Sgf1 restriction site is underlined)

reverse primer: 5'-CTCGCAGTTACTTCCAGTCCCTCAAAGCC-3'

Fragment 2

forward primer: 5'-GTCTTTGGGTCATGATCAACGAACCGG-3'

reverse primer: 5'-TAT<u>GTTTAAAC</u>TTTATAAATGGGTCAAAAAGAATTAGA-3'

(a PmeI restriction site is underlined)

Primers used for qRT-PCR

FOXP2:

forward primer: 5'-CCACGAAGACCTCAATGGTT-3'

reverse primer: 5'-TCACGCTGAGGTTTCACAAG-3'

ß-Actin

forward primer: 5'-CTACAATGAGCTGCGTGTGGC-3'

reverse primer: 5'-CAGGTCCAGACGCAGGATGGC-3'

siFOXP2 sequence used in transfection

siFOXP2: 5'-ATGGAAGACAATGGCATTAAA-3'

Primers used in miRNA binding sites mutagenesis

let7-a bs1-1#

5'-CCCAAAAGGCTTTCTGAAAGCTTCAAGCTCTGCAAAAAAAAAAAAAGAAAAA-3'

5'-TTTTTCTTTTTTTTTTTTTGCAGAGCTTGAAGCTTTCAGAAAGCCTTTTGGG-3'

let7-a bs1-2#

5'-CAAAAGGCTTTCTGAAAGCTTCAAGCACTGCAAAAAAAAAAAAAGAAAAAAAAA-3'

5'-TTTTTTTTTCTTTTTTTTTTTTTGCAGTGCTTGAAGCTTTCAGAAAGCCTTTTG-3'

let7-a bs2

5'-CTAGGATTTGAAAGAAAACTGTCAAGCACTTAACACCAGGGAGTTATCAGA-3'

5'-TCTGATAACTCCCTGGTGTTAAGTGCTTGACAGTTTTCTTTCAAATCCTAG-3'

miR-9 bs1

5'-CAGTCAAAGGCTACAGCTGCAACCTAAGGCCAACTCTAACCATGGC-3'

5'-GCCATGGTTAGAGTTGGCCTTAGGTTGCAGCTGTAGCCTTTGACTG-3'

miR-9 bs2-1#

5'-GTCCATGGTATTTATTTTCAGTCAAGCAAAAGTTACATATAATTCTGCCTCTG-3'

5'-CAGAGGCAGAATTATATGTAACTTTTGCTTGACTGAAAATAAATACCATGGAC-3'

miR-9 bs2-2#
5'-GTATTTATTTTCAGTCAAGCAAACGTTACATATAATTCTGCCTCTGC-3'
5'-GCAGAGGCAGAATTATATGTAACGTTTGCTTGACTGAAAATAAATAC-3'

miR-129-5p bs1-1#
5'-AAGGCTTTCTGAAAGCTTCTACCTCTGGAAAAAAAAAAAAAGAAAAAAAAAAAAAAG-3'
5'-CTTTTTTTTTTTTTCTTTTTTTTTTTTTCCAGAGGTAGAAGCTTTCAGAAAGCCTT-3'

miR-129-5p bs1-2#
5'-GGCTTTCTGAAAGCTTCTACCTCTGGATAAAAAAAAAAAGAAAAAAAAAAAAAAGAA-3'
5'-TTCTTTTTTTTTTTTTCTTTTTTTTTTTATCCAGAGGTAGAAGCTTTCAGAAAGCC-3'

miR-129-5p bs2
5'-CATGAACACTTGGCATATCTTACTTACGAAAAAAGAAGGATGTACATTTTACTA-3'
5'-TAGTAAAATGTACATCCTTCTTTTTTCGTAAGTAAGATATGCCAAGTGT-3'
